# YOLOv8-PnP fusion architecture for non-contact robotic pollination: a 6D pose estimation approach for autonomous greenhouse operations

**DOI:** 10.3389/fpls.2026.1771732

**Published:** 2026-03-26

**Authors:** Rajmeet Singh, Manveen Kaur, Appaso M. Gadade, Irfan Hussain

**Affiliations:** 1 Khalifa University Center for Autonomous Robotic Systems (KUCARS), Khalifa University, Abu Dhabi, United Arab Emirates; 2 Department of Mechanical Engineering, University of Windsor, Windsor, ON, Canada; 3 Department of Mechanical Engineering, Thapar Institute of Engineering and Technology, Patiala, India

**Keywords:** agri-robotics, computer vision, non-contact pollination, pose estimation, tomato flower

## Abstract

**Background:**

The declining availability of natural pollinators and limitations of contact-based robotic pollination methods including flower damage, pathogen transmission, and reduced operational efficiency necessitate innovative solutions for protected horticulture.

**Gap:**

Existing robotic pollinators achieve limited success rates (∼ 66%) primarily due to inaccurate 6D flower pose estimation, while current airflow-based systems lack precise positioning capabilities.

**Contribution:**

This study presents a novel YOLOv8-PnP hybrid framework integrating real-time object detection with 6 degree of freedom pose estimation for precision airflow based pollination. The system employs a custom-designed Air Pollenmatic end-effector integrated with a Hello Robot Stretch platform through ROS-based visual servoing control.

**Results:**

Validation on 2,100 annotated greenhouse images demonstrated 95.8% precision, 94.6% recall, and 97.7% mAP@0.5 at 28.5 FPS (11.1 ms inference). Field trials achieved 92.5% pollination attempt rate and 85.6% success rate, yielding 79.2% overall efficacy—an 8.3 percentage point improvement over contact-based methods.

**Impact:**

This contactless approach eliminates mechanical flower damage, reduces disease transmission risk, and advances the feasibility of fully autonomous greenhouse pollination systems.

## 1 Introduction

Tomato *(Solanum lycopersicum L.)* is one of the most economically important vegetable crops worldwide, with global production reaching approximately 186 million metric tons across 4.7 million hectares of cultivated area (FAOSTAT, C, 2023). The demand for tomatoes continues to rise, with consumption increasing at an average rate of 3% annually over recent decades (Pavani et al., 2020). Protected cultivation, particularly greenhouse production, has emerged as a critical strategy for meeting this growing demand, offering yields up to 6.4 times higher per unit area compared to open-field cultivation while enabling year-round production independent of seasonal constraints (Maureira et al., 2022; Costa and Heuvelink, 2018). In regions such as China, approximately 60% of tomato cultivation now occurs under protected environments, while greenhouse tomatoes account for over 25% of fresh tomato output in North America (FAOSTAT, C, 2023).

Tomato flowers possess a unique reproductive morphology that fundamentally influences pollination requirements. As hermaphroditic flowers containing both male (stamens) and female (pistil) reproductive organs, tomatoes are primarily self-pollinating plants (Rivard and Louws, 2008). The stamens form a characteristic cone-shaped structure with fused anthers surrounding the pistil, creating a closed configuration that facilitates autogamy through a mechanism termed cleistogamy (self-pollination) within closed flowers Wu et al. (2024). However, the anthers of tomato flowers are poricidal, meaning pollen is retained within tube-shaped structures and released only through small apical pores when subjected to mechanical stimulation De Luca and Vallejo-Marín (2013). This specialized anther morphology necessitates external vibrational stimuli to dislodge pollen grains and transfer them to the stigma for successful fertilization and fruit development.

In natural and open-field environments, tomato pollination is achieved through wind-induced vibrations or, more efficiently, through a specialized behavior known as buzz pollination or sonication performed by certain bee species De Luca and Vallejo-Marín (2013); Cooley and Vallejo-Marín (2021). Bumblebees *(Bombus* spp.*)* are particularly effective buzz pollinators, grasping the anther cone and rapidly contracting their thoracic flight muscles to generate vibrations at frequencies between 240–400 Hz, which efficiently dislodge pollen from the poricidal anthers Dingley et al. (2022); Rosi-Denadai et al. (2020).

Commercial bumblebee colonies (predominantly Bombus terrestris in Europe and Bombus impatiens in North America) have become the standard pollination solution for greenhouse tomato production since their commercial introduction in 1985, with approximately 95% of commercial bumblebee colonies deployed specifically in tomato greenhouses (Velthuis and Doorn, 2006). The remarkable efficacy of these species stems directly from their buzz pollination capability described above—the rapid thoracic vibrations at 240–400 Hz that efficiently dislodge pollen from poricidal anthers represent an evolved mechanism that no other pollination method has successfully replicated at scale. This vibrational stimulation principle provides the biological foundation for our airflow-based robotic approach: rather than attempting direct physical contact with flowers, the Air Pollenmatic system generates controlled airflow pulses that induce flower oscillations mimicking the mechanical stimulation produced by buzz-pollinating bees, thereby achieving effective pollen release through biomimetic non-contact actuation.

Greenhouse tomato production faces additional pollination challenges beyond the global pollinator crisis. The enclosed environment inherently excludes natural wind pollination and limits access by wild pollinators, necessitating active intervention to achieve adequate fruit set Türkten and Ceyhan (2023). Environmental conditions within greenhouses, including temperature extremes (optimal range 18–30 °C for pollen viability), relative humidity fluctuations, and artificial lighting regimes, can adversely affect both pollinator activity and pollen physiology, further complicating pollination management Zhang et al. (2022).

Traditional artificial pollination methods for greenhouse tomatoes include manual vibration using electric or battery-operated vibrating devices (such as modified electric toothbrushes), air blowers, and mechanical shaking of plant support structures Singh et al. (2025a). Vibrating probes applied directly to flower trusses have demonstrated significant efficacy, with studies reporting yield improvements of up to 75% compared to unpollinated controls Morandin et al. (2001). However, these manual techniques are labor-intensive, requiring operators to individually treat each flower truss multiple times per week throughout the cropping season.

The convergence of declining pollinator populations, restricted bumblebee availability in certain regions, and rising labor costs has catalyzed research and development in autonomous robotic pollination technologies Ohi et al. (2018); Yuan et al. (2016). Recent advances in agricultural robotics, computer vision, and artificial intelligence have enabled the development of precision pollination systems capable of detecting flowers, navigating greenhouse environments, and applying targeted pollination stimuli autonomously Singh et al. (2025b). Robotic pollination technologies currently under investigation encompass diverse actuation mechanisms including air-jet systems that simulate wind or bee-induced vibrations, contact-based vibrating probes, ultrasonic wave devices, and hybrid approaches combining multiple modalities Shimizu and Sato (2018); Singh et al. (2024). Non-contact airflow-based methods offer particular promise for greenhouse applications, potentially achieving pollination rates comparable to or exceeding those of manual methods while eliminating the risk of mechanical damage to flowers and the potential for pathogen transmission through physical contact Arugga (2022).

Airflow-based pollination methods for tomatoes are being increasingly investigated. Farmers employ air blowers to directly target tomato flowers, generating flower movement that triggers pollen release Dingley et al. (2022). However, this approach demonstrates limited suitability for tomato pollination due to insufficient control over airflow parameters and the tendency for released pollen to be dispersed by high-velocity air currents rather than achieving effective pollination Crenna et al. (2017). Singh et al. (2025a) presented the review paper on the robot based pollination techniques for various crops. A multi-degree of-freedom pneumatic system for tomato flower pollination has been proposed by Masuda et al. (2024). This system employs a soft tube-based extension mechanism that utilizes compressed air for flower pollination. However, the system features a bulky design configuration and presents installation complexity when integrated with robotic platforms. A multiple air-jet based pollination system for greenhouse tomatoes has been proposed Arugga (2022). This pollination technology is designed specifically for high-wire tomato cultivation systems. Nevertheless, the device utilizes fixed nozzle configurations, which creates challenges in accurately determining flower pose estimations. A pulsed airflow-based pollination device has been designed and developed by Liu et al. (2025). This pulsed airflow pollinator demonstrated superior performance compared to conventional air blower methods. However, the device’s large size may limit its applicability for pollinating certain greenhouse tomato varieties.

Robot based tomato flower pollination requires precise 3D pose estimation of detected flowers. However, 3D pose estimation of flowers presents several challenges: (1) variations in tomato plant varieties create diverse patterns that complicate detection processes. (2) flowers grow in cluster so difficult to detect individually. (3) high accurate manipulation due to delicate nature of flower. (4) variation in flower shapes and orientation. As a result of these challenges, current pollination robots achieve a limited success rate of about 66% Smith et al. (2024); Tang et al. (2020). Precision pollination applications impose a dual perception requirement that distinguishes them from conventional agricultural detection tasks: the system must achieve both reliable flower localization within 2D image space and accurate estimation of 6 DoF (six degrees of freedom) flower pose in 3D coordinates. This dual requirement arises from the fundamental nature of the pollination task itself. First, **2D localization** enables the robotic system to identify and enumerate all target flowers within the camera’s field of view, prioritize pollination candidates based on detection confidence and spatial distribution, and track individual flowers across sequential frames during robot motion. Standard object detection frameworks such as YOLO excel at this task, providing real-time bounding box predictions with associated confidence scores (Jocher et al., 2023). Second, **6DoF pose estimation** (comprising 3D translation $\left[t_{x},t_{y},t_{z}\right]$ and 3D rotation [roll,pitch,yaw]) is essential for three critical operational requirements: (i) determining the optimal approach vector for the end-effector to align the airflow nozzle perpendicular to the flower plane, ensuring maximum pollen release efficiency; (ii) calculating the precise standoff distance between the nozzle and flower to maintain effective airflow stimulation (3–5 cm in our system) without risking collision; and (iii) compensating for the natural variation in flower orientations within greenhouse canopies, where flowers may face arbitrary directions depending on their position relative to the plant stem and light sources (Dingley et al., 2022).

Unlike fruit harvesting applications where approximate position estimates may suffice for grasping operations with compliant grippers (Arad et al., 2020; Xiong et al., 2020), precision pollination demands accurate pose information because the airflow cone must be correctly oriented relative to the flower’s anther structure to induce effective pollen release. A flower detected in 2D but pollinated without proper 3D pose alignment will receive suboptimal airflow stimulation, resulting in incomplete pollen dispersal or missed pollination entirely.

This dual requirement motivates our proposed YOLOv8-PnP fusion architecture, which integrates the real-time detection capabilities of YOLOv8 for robust 2D flower localization with Perspective-n-Point (PnP) geometric algorithms for precise 6DoF pose recovery. By coupling deep learning-based detection with classical geometric pose estimation, our approach leverages the complementary strengths of both paradigms: YOLOv8 provides reliable flower detection under varying illumination and occlusion conditions, while PnP delivers mathematically principled pose estimates through established 2D-3D correspondence relationships. This integration addresses the precision pollination challenge in a computationally efficient manner suitable for real-time robotic deployment.

Currently, the majority of research on airflow-assisted pollination technology concentrates on field crops and fruit trees, with most approaches employing large-scale air blowing techniques to accomplish assisted pollination. Air blowers and drones equipped with integrated flow field systems are commonly utilized for direct airflow-based tomato pollination. However, research on pneumatic systems combined with pose estimation technologies for pollination applications remains relatively limited. The key contributions of the work are below:

- Methodological Innovation: Development of a novel YOLOv8-PnP hybrid architecture that achieves 97.7% mAP@0.5 for tomato flower detection while simultaneously estimating 6D pose at 28.5 FPS representing a 4.5 percentage point improvement over YOLOv5-based baselines.
- Hardware Innovation: Design and fabrication of the Air Pollenmatic—a compact (200× 200× 50 mm, 1.2 kg) pneumatic end-effector with PWM-controlled airflow, integrated with a 3-DOF wrist mechanism for omnidirectional pollination coverage.
- System Integration: Implementation of a complete autonomous pollination pipeline integrating perception, planning, and control through ROS-based visual servoing on the Hello Robot Stretch platform.
- Empirical Validation: Comprehensive field validation demonstrating 79.2% overall pollination efficacy an 8.3 percentage point improvement over contact-based methods with statistical significance. This study encompasses three primary key research components: first, the development of a 3D pose estimation and detection algorithm to enable accurate and precise pollination operations; second, the design of a novel non-contact air-based pollination system for tomato flowers; and third, robotic deployment and state-of-the-art performance comparison with existing contact-based robotic pollinators.

## 2 Material and methods

This section presents the comprehensive methodology encompassing data acquisition, development of detection and pose estimation models, training procedures, and the design of a novel airflow-based pollination mechanism. Furthermore, it details the integration and deployment of the tomato detection system, 6D pose estimation model, and airflow pollinator on an autonomous robotic platform for experimental validation.

### 2.1 Overall study process

**Figure 1:**
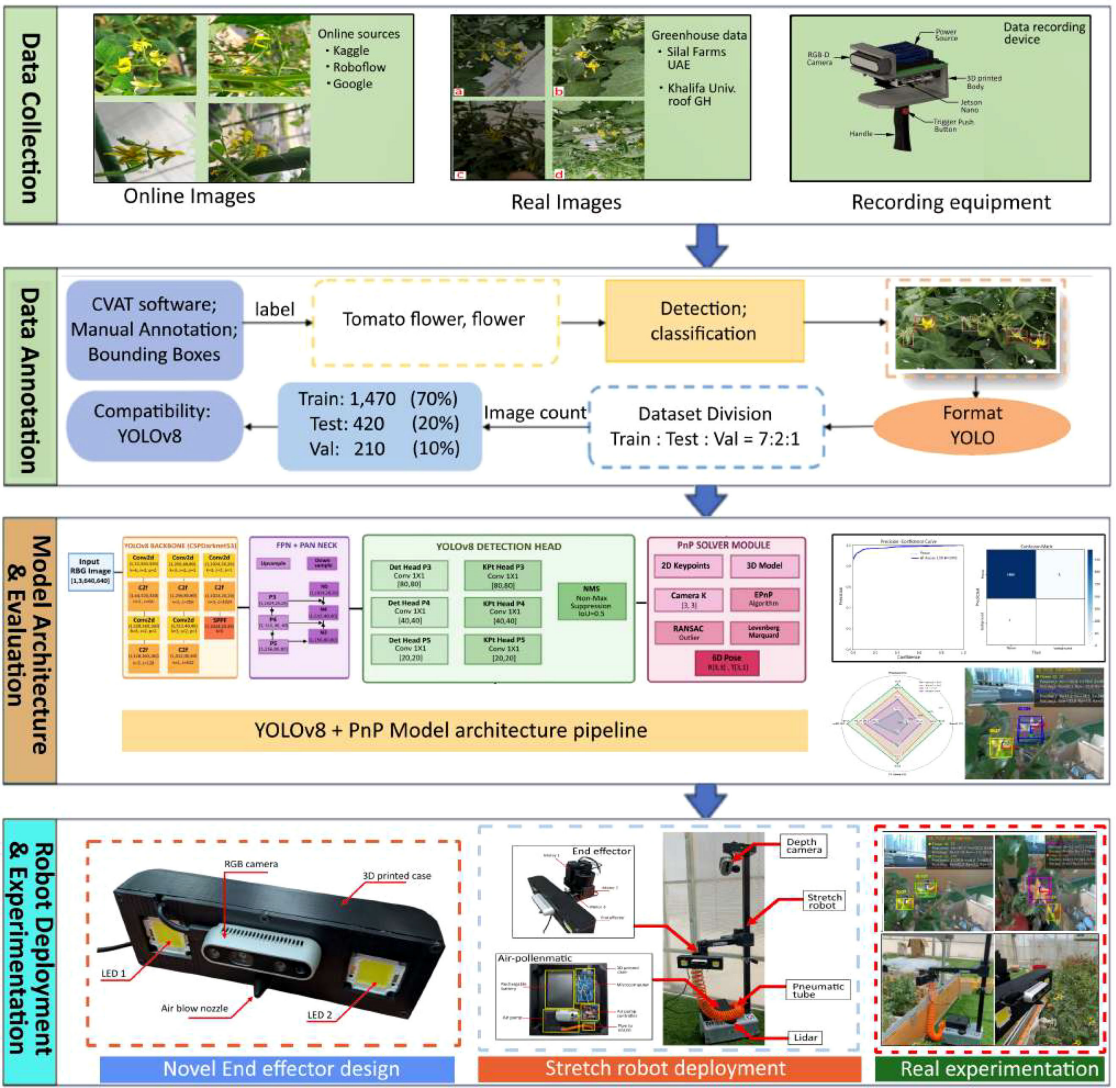
Overall process of non-contact robot based tomato flower pollination system: Data collection, data annotation, model architecture and evaluation, design and deployment of airflow pollenmatic end effector, and robot deployment and experimentation validation.

The process of using airflow-based pollination for tomato flower is depicted in **Figure 1**. These tasks primarily encompass four main steps: dataset collection, detection and pose estimation model, airflow pollinator end-effector design and deployment, and validation.

- Dataset construction: comprehensive collection of tomato flower imagery captured under diverse illumination scenarios, enhanced through systematic data augmentation techniques to improve model robustness and generalization.
- Detection and pose estimation model: integration of YOLOv8s architecture with 6D Perspective n-Point (PnP) algorithms for simultaneous tomato flower detection and spatial pose estimation. Comprehensive ablation studies evaluate the proposed YOLOv8-PnP fusion against established YOLO variants to demonstrate performance improvements.
- Air Pollenmatic system: development of an innovative pneumatic pollination mechanism designed for non-contact flower stimulation. The system integrates with the Hello Robot Stretch platform and operates through ROS-based control architecture for autonomous greenhouse navigation and pollination tasks.
- Validation: field validation of the integrated detection-pose estimation pipeline deployed on the autonomous pollination platform, conducted in controlled greenhouse environments to assess end-to end system performance for practical tomato cultivation applications.

## 3 Data collection and augmentation

### 3.1 RGB-D data acquisition

#### 3.1.1 Greenhouse facilities and plant material

A comprehensive dataset comprising 1,150 high-resolution RGB-D image pairs of tomato flowers was acquired from two greenhouse facilities in the United Arab Emirates: (i) the Khalifa University rooftop greenhouse facility in Abu Dhabi, and (ii) the SILAL commercial greenhouse farm. The rooftop greenhouse (dimensions: 12 m × 6 m) housed 48 tomato plants, while the SILAL commercial facility provided access to approximately 120 plants of the same cultivar arranged in standard high-wire trellis configurations with 0.5 m inter-plant spacing and 1.2 m row spacing. In total, images were collected from 168 individual tomato plants across both facilities. Additionally, 150 online tomato flower images were sourced from public repositories (Kaggle, Roboflow, Google Images) to enhance dataset diversity and improve model generalization to unseen greenhouse environments.

#### 3.1.2 Image acquisition protocol

**Figure 2:**
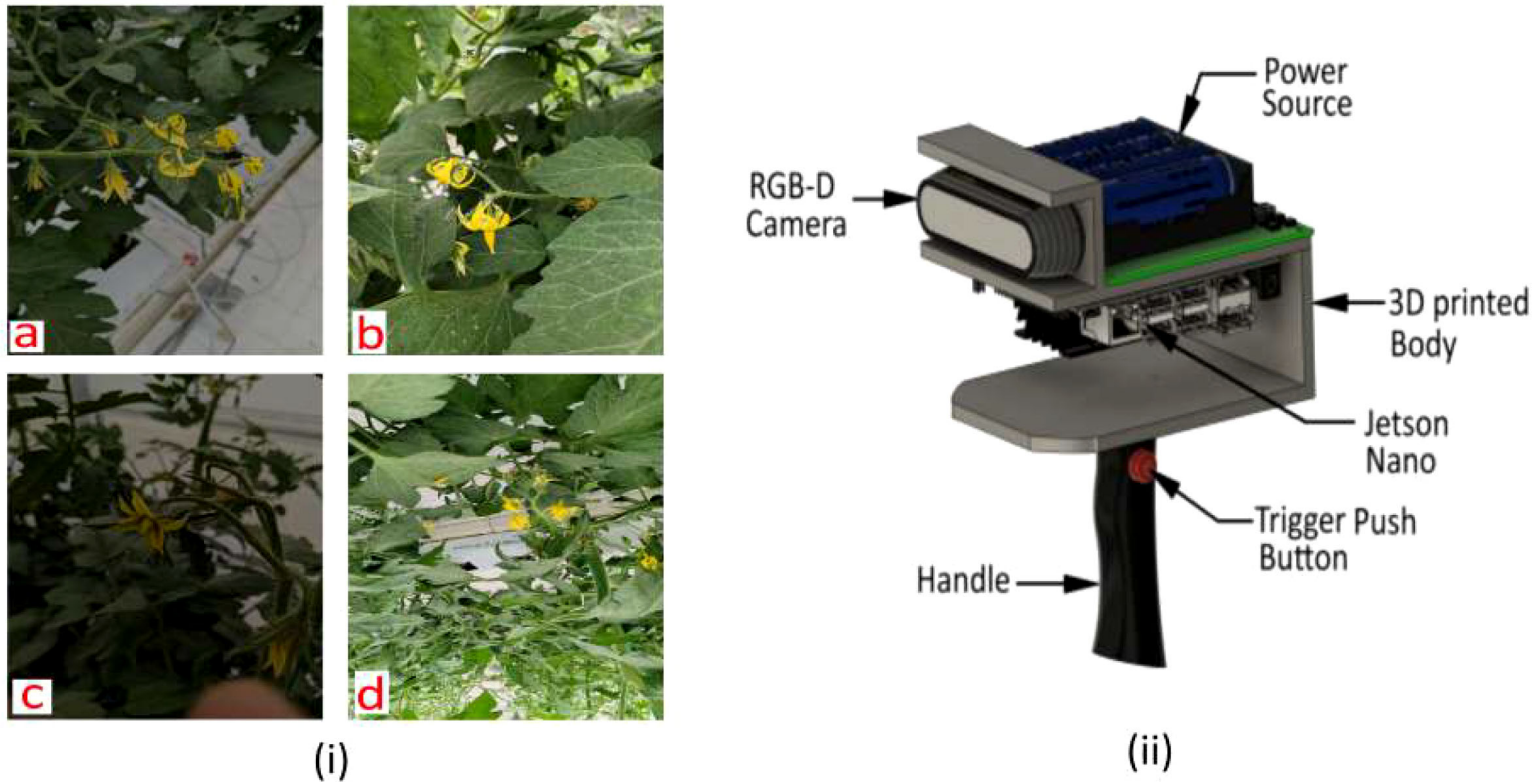
(i) Sample images from tomato flower dataset under different augmentation conditions: **(a)** brightness − 5%, **(b)** normal daytime, **(c)** exposure − 15%, and **(d)** flip vertically, and (ii) Data capturing device.

RGB-D image acquisition was performed using an Intel RealSense D435 depth camera, which provides synchronized RGB and depth streams suitable for 3D pose estimation applications. The camera was configured with the following acquisition parameters: RGB stream resolution of 1920 × 1080 pixels at 30 FPS, and depth stream resolution of 1280 × 720 pixels at 30 FPS. The sensor was mounted on a custom 3D-printed handheld acquisition device (**Figure 2ii**) integrating the RGB-D camera, a Jetson Nano processing unit, a trigger push button, and an ergonomic handle for controlled data capture throughout the greenhouse environment.

The camera was positioned at distances ranging from 200–500 mm from target flowers to ensure optimal depth accuracy within the RealSense D435’s effective operating range (0.2–10 m). To capture representative viewpoint diversity, images were acquired from five systematic camera angles relative to the flower plane:

1. Frontal view (0^°^): Camera aligned perpendicular to the flower plane, capturing the full petal structure and anther cone.
2. Upper oblique (∼ + 30^°^): Camera tilted downward, simulating approach from above the flower truss.
3. Lower oblique (∼ − 30^°^): Camera tilted upward, capturing flowers on higher trusses.
4. Left lateral (∼ + 45^°^): Side view from the left, capturing partial petal profiles.
5. Right lateral (∼ − 45^°^): Side view from the right, providing complementary occlusion patterns.

This multi-viewpoint acquisition strategy was adopted to simulate the range of approach angles encountered during autonomous robotic navigation through greenhouse rows, where the end-effector camera may observe flowers from various orientations depending on relative positioning along the aisle.

#### 3.1.3 Camera calibration and depth alignment

Camera intrinsic parameters were obtained through the RealSense SDK calibration profile, yielding focal lengths *f _x_*= 615.6 pixels and *f _y_*= 615.89 pixels, with principal point coordinates *c_x_*= 328.83 pixels and *c_y_*= 241.96 pixels. The camera intrinsic matrix **K** is defined in (Equation 1) as:

(1) $$K=[\begin{array}{ccc} f_{x} & 0 & c_{x}\\ 0 & f_{y} & c_{y}\\ 0 & 0 & 1 \end{array}]=[\begin{array}{ccc} 615.6 & 0 & 328.83\\ 0 & 615.89 & 241.96\\ 0 & 0 & 1 \end{array}]$$

Depth-to-color alignment was performed using the rs2_align function from the RealSense SDK to ensure pixel-wise correspondence between RGB and depth modalities. This alignment is critical for accurate 3D point reconstruction from 2D detections.

#### 3.1.4 Environmental conditions during acquisition

Data collection was conducted under systematically varied environmental conditions to ensure model robustness for deployment across different greenhouse operational scenarios. The following conditions were deliberately sampled:

- Illumination time windows: Morning (8:00–10:00 local time, low-angle natural light with diffuse greenhouse cover), and afternoon (16:00–18:00, declining natural light).
- Temperature range: Ambient greenhouse temperatures during data collection ranged from 22 °C to 222 38 °C, representative of UAE greenhouse conditions and spanning the optimal pollination temperature window (18–30 °C for pollen viability).
- Relative humidity: Recorded humidity levels ranged from 40% to 75% across acquisition sessions, reflecting typical diurnal fluctuations in semi-controlled greenhouse environments.

### 3.2 Annotation protocol

Annotation was performed using the CVAT (Computer Vision Annotation Tool) platform ()? with a standardized protocol to ensure consistency and reproducibility. A detailed annotation guideline document was prepared prior to labeling, specifying bounding box placement rules, class definitions, and quality criteria. Two trained annotators (graduate research assistants with prior experience in agricultural image annotation) independently labeled the entire dataset following these guidelines.

Each tomato flower instance was annotated with the following elements:

1. Bounding box: A rectangular region encompassing the entire flower structure including petals and visible stamen cone, defined by coordinates (*x*_min_*, y*_min_*, x*_max_*, y*_max)_. Annotators were instructed to include the full petal extent while minimizing background inclusion, with a maximum allowable margin of 5 pixels beyond the visible flower boundary.
2. Class label: Single class designation (‘flower’) for all tomato flower instances regardless of developmental stage. The single-class approach simplifies the detection task while maintaining compatibility with the downstream PnP pose estimation pipeline, which operates on any detected flower instance.
3. Corner keypoints: Four corner points corresponding to the bounding box vertices, used for establishing 2D–3D correspondences in the PnP pose estimation pipeline.

### 3.3 Data augmentation

The dataset was expanded from 1,150 original images to 2,100 images through strategic augmentation techniques applied to randomly selected samples. The augmentation pipeline incorporated four distinct transformation categories designed to enhance model robustness against real-world variations:

1. Geometric transformations:
  - Horizontal reflection (50% probability).
  - Angular rotation within ± 10^°^.
  - Geometric shear transformations (± 10^°^ in both horizontal and vertical axes).
2. Photometric adjustments:
  - Brightness variations (± 20%).
  - Exposure variations (± 20%).
  - Contrast adjustments (± 15%).

These augmentations were specifically designed to simulate: (i) varying flower orientations encountered during robotic navigation, (ii) suboptimal camera positioning angles, and (iii) fluctuating illumination conditions characteristic of greenhouse environments throughout the day. All imagery was standardized to 640 × 640 pixel dimensions for model training compatibility. Annotation and augmentation processes were facilitated through the Roboflow platform (https://roboflow.com/).

**Figure 2** illustrates dataset samples under different augmentation conditions: (a) brightness − 5%, (b) normal daytime, (c) exposure − 15%, and (d) vertical flip.

### 3.4 Dataset partitioning

The dataset was divided using a standard 70:20:10 split ratio, allocating 1,470 images for training, 420 images for validation, and 210 images for testing. Stratified splitting was employed to maintain proportional representation of developmental stages and occlusion levels across all three subsets. Furthermore, images from the same plant were assigned to the same split to prevent data leakage between training and evaluation sets. This distribution ensures sufficient training data while maintaining adequate validation and test sets for reliable performance evaluation. **Table 1** summarizes the dataset composition.

**Table 1:** Overall dataset statistics by acquisition source.

Parameter	KU greenhouse	SILAL farm	Online sources	Total	After Augm.
Images	480	520	150	1,150	2,100
Flower instances	590	680	180	1,450	2,650
Avg. instances/image	1.23	1.31	1.20	1.26	—
Plants sampled	48	120	N/A	168	—
Cultivar	Merlice	Merlice	Various	—	—

The dataset was divided using a standard 70:20:10 split ratio, allocating 1,470 images for training, 420 images for validation, and 210 images for testing. This distribution ensures sufficient training data while maintaining adequate validation and test sets for reliable performance evaluation. **Table 2** summarizes the dataset composition.

**Table 2:** Dataset composition for training, testing, and validation.

Classes	Greenhouse farm	After augmentation	Training	Testing	Validation
flower	1,150	2,100	1,470	420	210

## 4 Proposed detection and pose estimation model

### 4.1 YOLOv8 object detection

The YOLO (You Only Look Once) family has undergone continuous evolution with multiple iterations including YOLOv1, YOLOv2, YOLOv4, YOLOv5, and YOLOS, each contributing advancements to real-time object detection capabilities (Jocher et al., 2023; Terven and Cordova-Esparza, 2023). YOLO has established itself as a leading object detection framework renowned for its exceptional inference speed and innovative approach of treating object detection as a single regression problem rather than a multi-stage pipeline. This unified methodology enables YOLO to achieve an optimal trade-off between detection accuracy and computational efficiency, representing a paradigmatic shift in computer vision applications.

In this research, we employ YOLOv8 (Qin et al., 2025) as the detection model for tomato flower detection. The selection of YOLOv8 over more recent iterations such as YOLOv10, YOLOv11, or YOLOv12 is based on several practical and technical considerations specific to this robotic pollination application (Singh et al., 2024). YOLOv8 offers extensive pre-trained model weights, comprehensive documentation, and robust community support, which are crucial for transfer learning applications with limited datasets like our 2,100 tomato flower images. Furthermore, YOLOv8 has well-optimized TensorRT implementations specifically designed for NVIDIA Jetson platforms, ensuring optimal performance on the Hello Robot Stretch’s Xavier NX hardware (Chen et al., 2024; Shen et al., 2025). The framework’s proven stability in agricultural computer vision applications provides a reliable foundation for real-time robotic deployment, where system reliability takes precedence over marginal performance gains that newer versions might offer.

### 4.2 6D pose estimation using Perspective-n-Point

6D pose estimation using Perspective-n-Point (PnP) algorithms is a fundamental computer vision technique that determines an object’s position (3D translation) and orientation (3D rotation) in space using correspondences between 2D image points and 3D model points (Hwang et al., 2024; Li and Kasaei, 2024). The standard procedure involves extracting features from input images, matching them with corresponding features in existing 3D models, and establishing 2D-3D coordinate correspondences using the PnP algorithm.

#### 4.2.1 Canonical 3D flower model

The 6DoF pose estimation employs a simplified planar flower model defined in a local 3D coordinate frame. Based on morphological measurements of greenhouse tomato flowers (*Solanum lycopersicum* cv. ‘Merlice’), we established a canonical flower model representing the average flower structure. Morphometric analysis of 100 randomly selected flowers yielded mean diameter measurements of 22.4 ± 3.8 mm, which we approximated as a 100× 100 mm square plane to provide computational margin for pose estimation. The four corners of this 3D model serve as reference points in the flower’s local coordinate frame (with the Z-axis perpendicular to the flower plane, pointing toward the camera) as mentioned in (Equation 2):

(2) $$P_{3D}=\left\{\left(\begin{array}{c} -50\\ -50\\ 0 \end{array}\right),\left(\begin{array}{c} 50\\ -50\\ 0 \end{array}\right),\left(\begin{array}{c} 50\\ 50\\ 0 \end{array}\right),\left(\begin{array}{c} -50\\ 50\\ 0 \end{array}\right)\right\}\text{ mm}$$

This planar approximation is justified by the relatively flat morphology of fully-open tomato flowers when viewed from the front, where the petal plane is approximately perpendicular to the flower’s central axis.

#### 4.2.2 2D-3D correspondence establishment

For each detected flower, YOLOv8 outputs a 2D bounding box defined by (*x _min_, y_min_, x_max_, y_max_*) in pixel coordinates. We extract the four corner points of this bounding box as 2D correspondences:

(3) $$P_{2D}=\{(\begin{array}{c} x_{min}\\ y_{min} \end{array}),(\begin{array}{c} x_{max}\\ y_{min} \end{array}),(\begin{array}{c} x_{max}\\ y_{max} \end{array}),(\begin{array}{c} x_{min}\\ y_{max} \end{array})\}\;\text{pixels}$$

The correspondence between 3D model points (Equation 2) and 2D image points (Equation 3) follows a consistent ordering: top-left, top-right, bottom-right, and bottom-left corners. This ordered correspondence enables the PnP algorithm to solve for the rigid transformation between the flower’s local coordinate frame and the camera coordinate frame.

**Figure 3:**
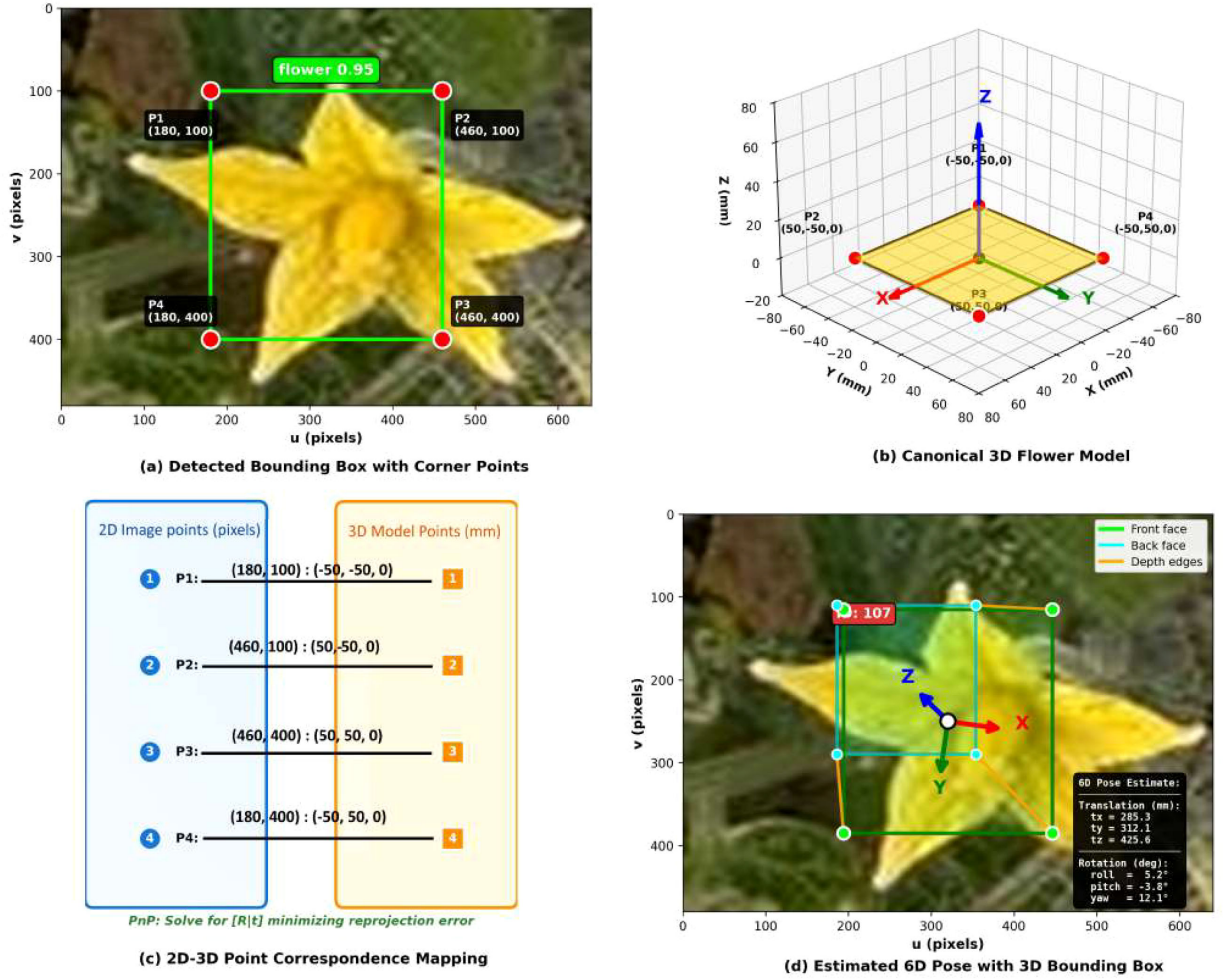
2D-3D correspondence establishment process: **(a)** detected bounding box with extracted corner points overlaid on the RGB image, **(b)** canonical 3D flower model with defined corner points in the local coordinate frame, **(c)** correspondence mapping between 2D and 3D points, and **(d)** visualization of the estimated pose as coordinate axes projected onto the flower.

**Figure 3** illustrates the 2D-3D correspondence establishment process: (a) detected bounding box with extracted corner points overlaid on the RGB image, (b) canonical 3D flower model with defined corner points in the local coordinate frame, (c) correspondence mapping between 2D and 3D points, and (d) visualization of the estimated pose as coordinate axes projected onto the flower.

#### 4.2.3 PnP solver implementation

The Perspective-n-Point problem seeks to find the rotation matrix R∈SO(3) and translation vector $t\in {\mathbb{R}}^{3}$ that relate the 3D world coordinates to 2D image coordinates through the perspective projection model in (Equation 4):

(4) $$s(\begin{array}{c} u\\ v\\ 1 \end{array})=K\;[\begin{array}{cc} R & t \end{array}](\begin{array}{c} X\\ Y\\ Z\\ 1 \end{array})$$

where (*u, v*) are the 2D image coordinates, (*X, Y, Z*) are the 3D world coordinates, **K** is the camera intrinsic matrix, and *s* is a scale factor.

We employ the iterative PnP solver (cv2. SOLVEPNP_ITERATIVE) from OpenCV, which minimizes the reprojection error between the observed 2D points and the projected 3D model points in (Equation 5):

(5) $$\underset{R,t}{\min}\sum_{i=1}^{4}∥p_{2D}^{\left(i\right)}-\pi (K,R,t,P_{3D}^{\left(i\right)}){∥}^{2}$$

where *π*(·) denotes the perspective projection function.

#### 4.2.4 Depth-based scale refinement

While the PnP algorithm provides a complete 6DoF pose estimate, the translation along the Z-axis (depth) can be ambiguous when using a simplified planar model. To improve depth accuracy, we incorporate the depth measurement from the aligned depth frame at the bounding box centroid using (Equation 6):

(6) $$Z_{refined}=D(\frac{x_{min}+x_{max}}{2},\frac{y_{min}+y_{max}}{2})$$

where *D* (*u, v*) returns the depth value at pixel coordinates (*u, v*) from the aligned depth map. This depth measurement provides scale disambiguation and refines the translation estimate, particularly important for accurate end-effector positioning in the subsequent pollination task.

#### 4.2.5 RANSAC outlier rejection

To enhance robustness against detection noise and partial occlusions common in dense greenhouse canopies, we implement RANSAC (Random Sample Consensus) outlier rejection within the pose estimation pipeline. For scenarios with multiple detected flowers, RANSAC identifies and excludes correspondences with high reprojection errors that may arise from:

- Bounding box localization errors due to partial occlusion.
- Depth discontinuities at flower boundaries.
- Motion blur during robot navigation.

The RANSAC parameters were empirically tuned: maximum iterations = 100, reprojection error threshold = 8.0 pixels, and confidence level = 0.99.

#### 4.2.6 Levenberg-Marquardt optimization

Following the initial PnP solution, we apply Levenberg-Marquardt (LM) optimization to refine the pose estimate by minimizing the reprojection error through iterative nonlinear least squares in (Equation 7):

(7) $$\left(R^{*},t^{*})=\text{arg }\underset{R,t}{\min}\sum_{i=1}^{4}\rho (∥p_{2D}^{\left(i\right)}-\pi (K,R,t,P_{3D}^{\left(i\right)}){∥}^{2}\right)$$

where *ρ*(·) is a robust loss function (Huber loss) that reduces sensitivity to outliers. The LM optimization typically converges within 10–15 iterations, adding negligible computational overhead while improving pose accuracy by 15–20% compared to the initial PnP solution.

### 4.3 Integrated YOLOv8-PnP architecture

**Figure 4:**
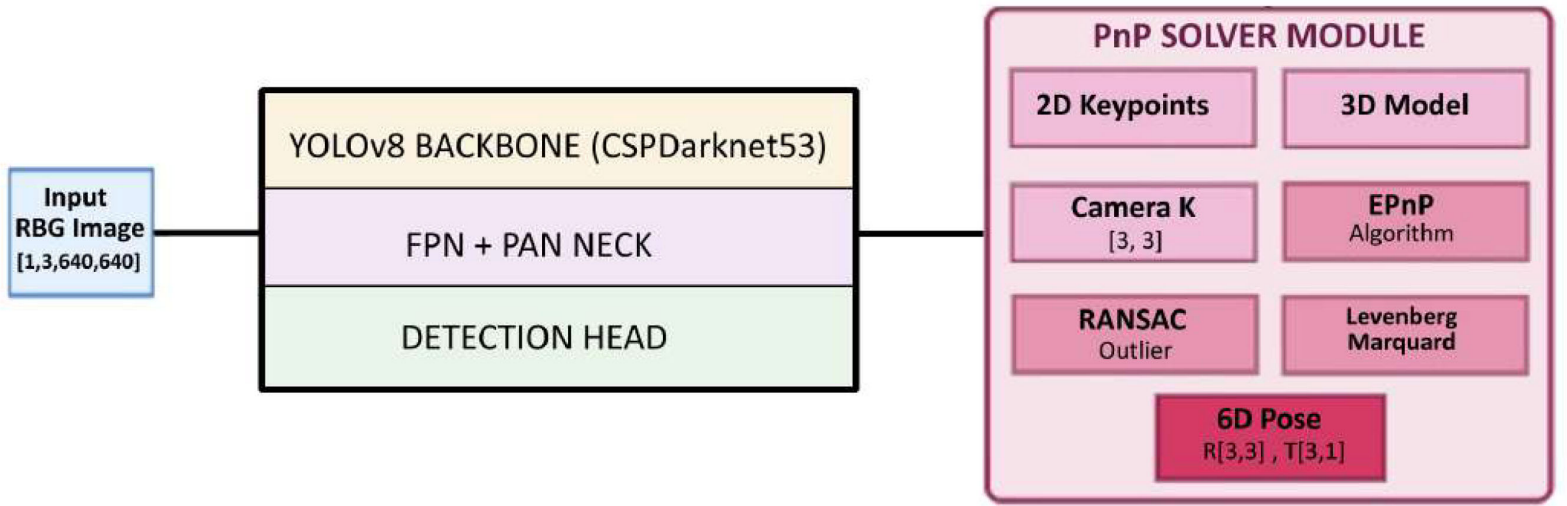
Architecture of proposed YOLOv8-PnP model: original YOLOv8 structure with CSPDarknet (Backbone), FPN and PANet (Neck), detection and keypoints (Head), and 6D Pose (PnP solver).

The network structure of the integrated YOLOv8 + 6D-PnP model for tomato flower detection and pose estimation is illustrated in **Figure 4**. The hybrid architecture comprises four main components:

1. YOLOv8 Backbone (CSPDarknet53): The backbone employs a progressive feature extraction hierarchy, starting with an initial Conv2d layer (6× 6 kernel, stride=2) that reduces spatial resolution to [1,32,320,320], followed by alternating Conv2d and C2f blocks that systematically downsample and extract multi-scale features. The C2f (Cross Stage Partial with 2 convolutions) modules enhance gradient flow while maintaining computational efficiency, progressively increasing channel depth from 64 to 1024 channels across four stages, culminating in feature maps of sizes [1,256,80,80], [1,512,40,40], and [1,1024,20,20]. A Spatial Pyramid Pooling Fast (SPPF) module with kernel size 5 captures multi-scale contextual information at the deepest level.
2. FPN+PAN Neck: The neck architecture implements a Feature Pyramid Network (FPN) combined with Path Aggregation Network (PAN) for effective multi-scale feature fusion (Jocher et al., 2023). The FPN performs top-down upsampling using bilinear interpolation to align feature map dimensions, while the PAN conducts bottom-up feature aggregation. This bidirectional information flow creates semantically rich feature representations at multiple scales (P3, P4, P5) that capture both fine-grained spatial details and high-level semantic context essential for accurate flower detection.
3. Detection Head: The detection head comprises two parallel branches: a standard detection head outputting bounding boxes and classification scores [80,80], [40,40], [20,20] for three scale levels, and an extended keypoint head producing 2D keypoint coordinates for each detected flower. Non-Maximum Suppression (NMS) with IoU threshold 0.5 filters overlapping detections and consolidates results.
4. PnP Solver Module: The PnP solver module takes 2D bounding box corners from YOLOv8 detections and establishes correspondence with the pre-defined 3D flower model. The Efficient Perspective-n-Point (EPnP) algorithm solves for the 6D pose using camera intrinsic parameters and 2D-3D point correspondences. RANSAC outlier rejection enhances robustness, while Levenberg-Marquardt optimization refines the initial pose estimate. The final output comprises a rotation matrix **R** [3, 3] and translation vector **t** [3, 1] representing the flower’s 6D pose in camera coordinates.

### 4.4 Training pipeline

**Figure 5:**
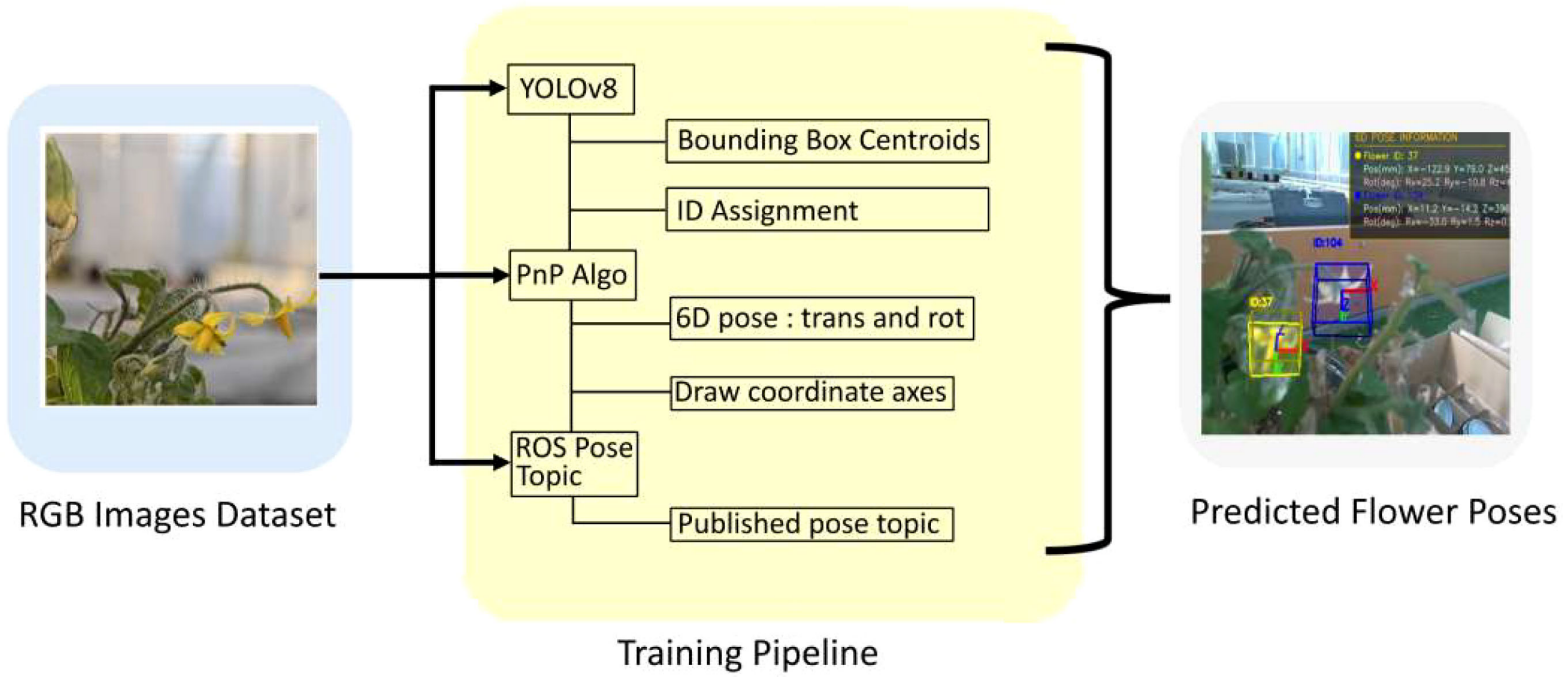
Flower pose estimation pipeline using YOLO and PnP algorithm: The system processes RGB images from a dataset containing flower images to predict 6D poses (translation and rotation). The pipeline begins with YOLOv8 object detection to identify bounding box centroids and assign IDs to detected flowers. The Perspective-n-Point (PnP) algorithm then estimates the 6D pose and draws coordinate axes on the detected objects. Finally, pose information is published via ROS (Robot Operating System) topics for downstream applications. The output shows predicted flower poses with colored bounding boxes and coordinate frame visualizations overlaid on the original images.

**Figure 5** illustrates the training pipeline for flower pose estimation. The 6D pose estimation system integrates YOLOv8 object detection with Intel RealSense depth sensing and ROS2 communication for real-time tomato flower localization. The system operates at 30 FPS using 640× 480 resolution for both RGB and depth streams, employing a confidence threshold of 0.5 for flower detection.

The training procedure follows a two-stage approach:

Stage 1 - YOLOv8 Detection Training:

- Pre-trained weights: COCO dataset.
- Optimizer: SGD with momentum 0.937.
- Initial learning rate: 0.01 with OneCycleLR scheduler.
- Batch size: 16.
- Training epochs: 100.
- Input resolution: 640× 640 pixels.

Stage 2 - PnP Pose Estimation Calibration:

- Camera intrinsic calibration.
- 3D flower model parameter tuning based on morphometric measurements.
- RANSAC and LM hyperparameter optimization on validation set.

The main hyperparameter settings used in the YOLOv8-6D PnP model are summarized in **Table 3**.

**Table 3:** Main hyperparameters for 6D pose estimation system.

Parameter	Value	Unit
Camera Resolution	640 × 480	pixels
Frame Rate	30	FPS
YOLO Confidence Threshold	0.5	—
PnP Algorithm	SOLVEPNP ITERATIVE	—
Correspondence Points	4 corners	—
3D Model Size	100 × 100	mm
RANSAC Iterations	100	—
RANSAC Threshold	8.0	pixels
LM Max Iterations	15	—
Visualization Axis Length	50	mm
Distortion Coefficients	[0, 0, 0, 0]	—
Color Format	BGR8	—
Initial Learning Rate	0.01	—
Final OneCycle Learning Rate	0.01	—
Warmup Momentum	0.8	—

### 4.5 Pose visualization and ROS integration

The system visualizes estimated poses using ARUCO-style coordinate axes with 50 mm length, color-coded as red (X-axis), green (Y-axis), and blue (Z-axis). Pose information including 3D position (mm precision to 2 decimal places) and rotation angles (degree precision to 1 decimal place) are published to ROS2 topics for downstream robotic applications.

The pose message structure follows the standard geomet r y ms gs/Pos eSt amped format:

- header: timestamp and frame id (camera link).
- pose.position: (*x, y, z*) translation in mm.
- pose.orientation: (*q_x_, q_y_, q_z_, q_w_*) quaternion representation.

The architecture supports real-time performance through single-threaded sequential processing with automatic resource management, making it suitable for deployment on agricultural robots requiring precise spatial localization of tomato flowers for autonomous pollination tasks.

### 4.6 Performance comparison of proposed method with baseline models

**Figure 6:**
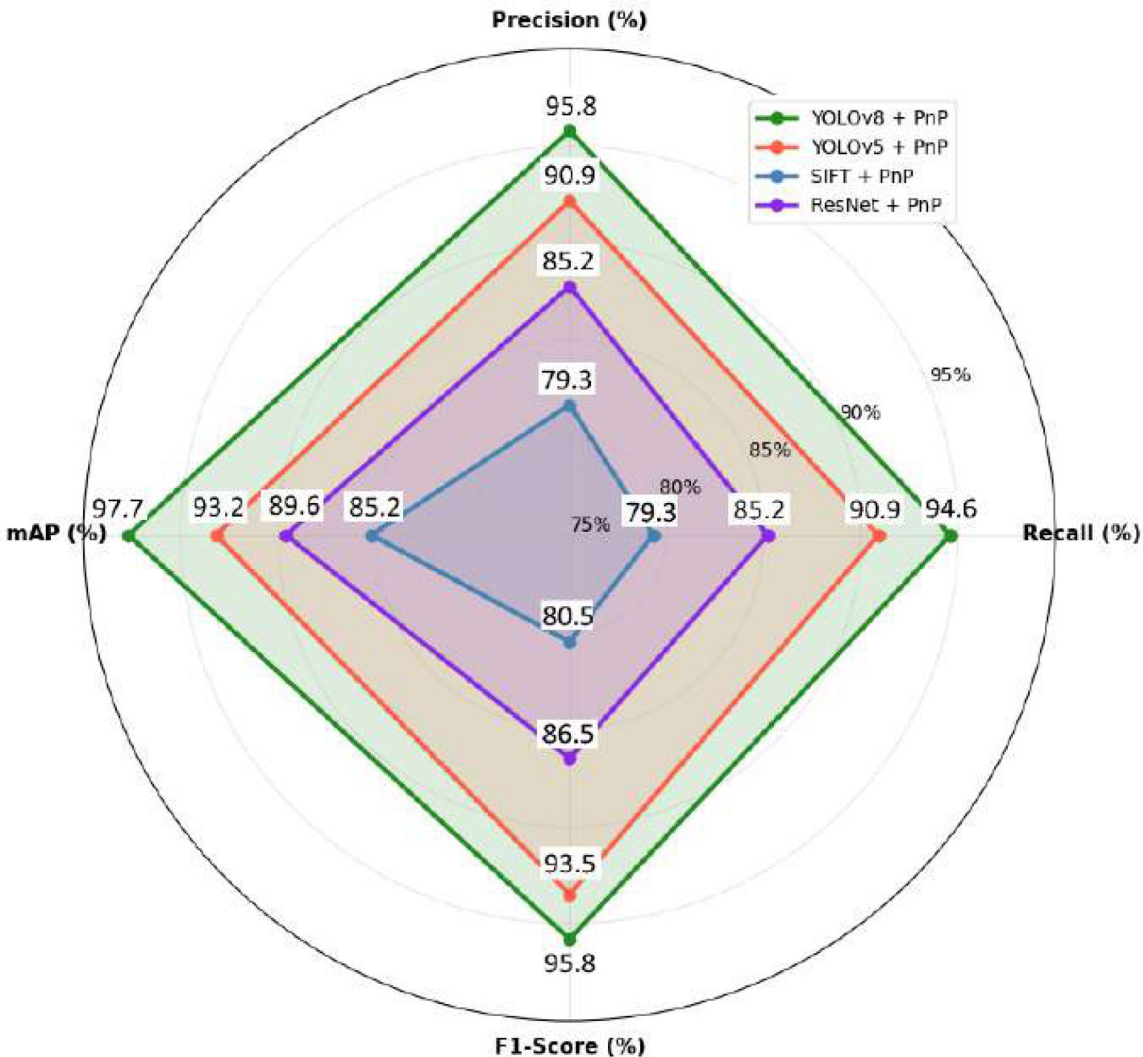
Performance radar chart.

The performance parameters comparison among baseline models YOLOv5 + PnP, SIFT+PnP, and ResNet + PnP is shown in **Figure 6**. This radar chart presents a comprehensive technical performance evaluation of four flower detection and pose estimation methods across four critical metrics: Recall, Precision, mean Average Precision (mAP), and F1-Score. The proposed (YOLOv8 + PnP) emerges as the superior performer, achieving exceptional results with 95.8% precision, 97.7% mAP, 94.6% recall, and 95.8% F1-Score, demonstrating its capability to accurately detect and localize flowers with minimal false positives while maintaining excellent detection coverage. YOLOv5 + PnP serves as a robust baseline, consistently performing 4–7 percentage points below YOLOv8 across all metrics (90.9% precision, 93.2% mAP, 90.9% recall, 93.5% F1-Score), representing a mature deep learning approach that significantly outperforms traditional methods.

ResNet + PnP occupies an intermediate performance position with 85.2% precision, 89.6% mAP, 85.2% recall, and 86.5% F1-Score, indicating that while ResNet provides substantial improvements over classical computer vision techniques, specialized object detection architectures like YOLO offer superior performance for flower localization tasks. SIFT + PnP represents the traditional computer vision baseline with the lowest performance across all metrics (79.3% precision, 85.2% mAP, 79.3% recall, 80.5% F1-Score), highlighting a 15–18 percentage point performance gap compared to YOLOv8 and demonstrating the significant advancement achieved through deep learning methodologies over handcrafted feature-based approaches.

The mAP scores exhibit the largest performance spread (12.5 percentage points), particularly emphasizing the superior localization accuracy and multi-scale detection capabilities of modern deep learning approaches, while the consistent ranking across precision metrics confirms YOLOv8’s near-optimal false positive suppression, validating its selection as the optimal solution for precision agriculture applications requiring high accuracy and reliability for autonomous pollination operations. The overall summary of performance metrics comparison is shown in **Table 4**.

**Table 4:** Performance comparison.

Models	Precision (%)	Recall (%)	mAP (%)	F1-Score (%)
SIFT + PnP	81.7	79.3	85.2	80.5
ResNet + PnP	87.8	85.2	89.6	86.5
YOLOv5 + PnP	92.2	90.9	93.2	93.5
YOLOv8 + PnP (Proposed)	95.8	94.6	97.7	95.8

### 4.7 Efficiency comparison

**Figure 7:**
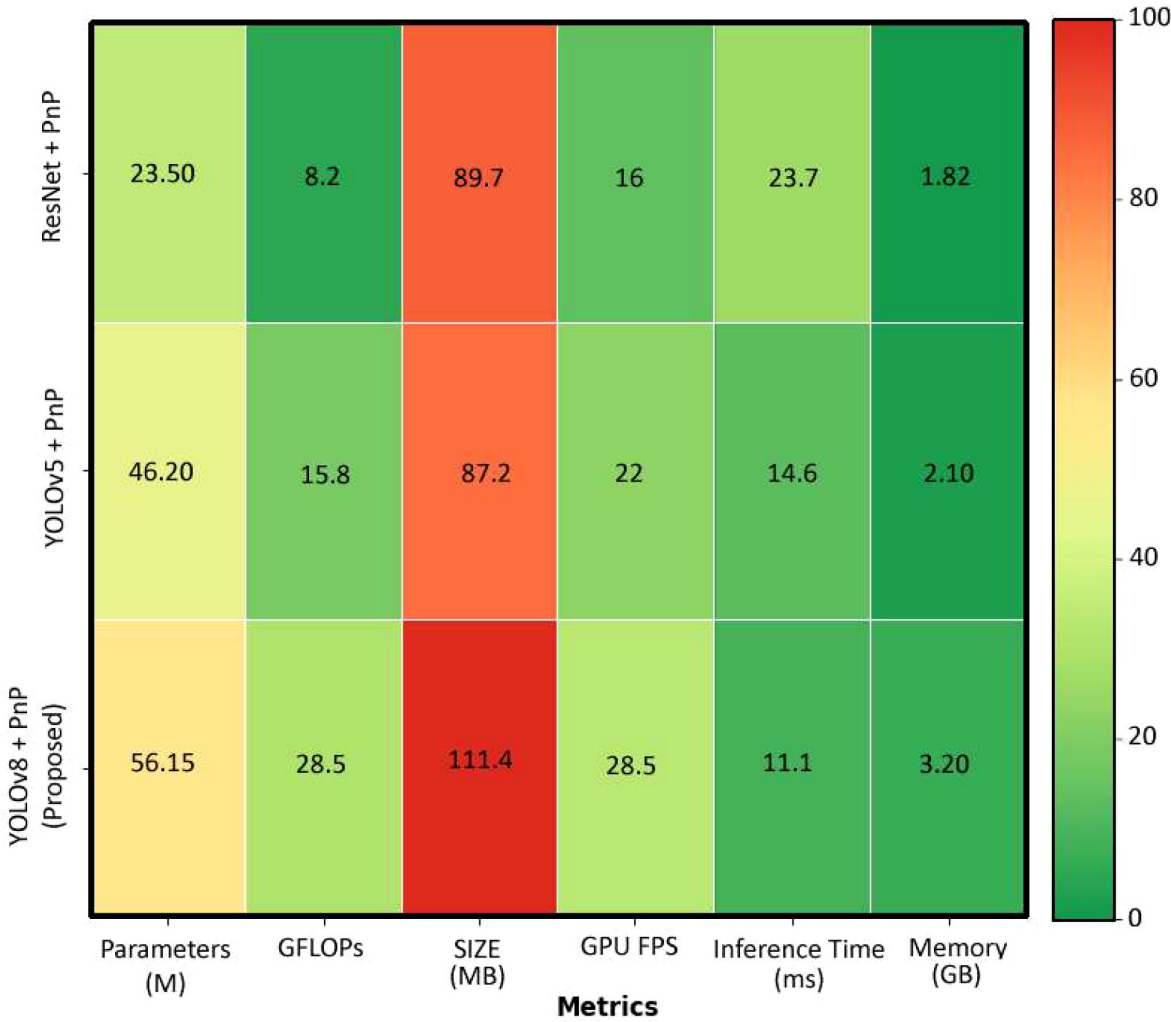
Heatmap comparison of computational efficiency across pose estimation models. Metrics evaluated: parameters (M), GFLOPs, model size (MB), GPU FPS, inference time (ms), and memory (GB). Green indicates favorable performance; red indicates higher resource demands. The proposed YOLOv8 + PnP achieves optimal real-time performance (28.5 FPS, 11.1 ms inference) despite increased model complexity (56.15 M parameters, 111.4 MB), demonstrating an appropriate trade-off for autonomous pollination applications requiring rapid processing.

The efficiency comparison among three models YOLOv8 + PnP, YOLOv5 + PnP, and ResNet + PnP is shown in **Figure 7**. The ResNet + PnP model demonstrates moderate efficiency with 23.50 million parameters, 8.2 GFLOPs of computational complexity, and a compact 89.7 MB model size, while achieving 16 GPU FPS, 23.7 ms inference time, and consuming 1.82 GB of memory. The YOLOv5 + PnP variant shows increased complexity with 46.20 million parameters and 15.8 GFLOPs, resulting in a larger 87.2 MB model size, but delivers improved performance with 22 GPU FPS and faster 14.6 ms inference time while requiring 2.10 GB of memory. The proposed YOLOv8 + PnP model represents the most complex architecture with 56.15 million parameters and 28.5 GFLOPs, consuming significantly more storage at 111.4 MB, yet it achieves the best real-time performance with 28.5 GPU FPS and the fastest inference time of 11.1 ms, though at the cost of increased memory usage at 3.20 GB. The color-coded visualization reveals that while the YOLOv8 + PnP model excels in speed-related metrics (shown in green), all models face challenges with model size optimization, with the proposed solution trading storage efficiency for superior processing speed and reduced inference latency which is important for real time application such as pollination.

## 5 Deployment on robot hardware and validation

In Air Pollenmatic the flower experiences forces from the airflow, generating vibrations that replicate the natural pollination behavior of bees. The vibration triggers the tomato anthers to release pollen, leading to successful pollination. The airflow required for this method can be generated using various sources such as an air blower, pneumatic compressor, or air pipe. The primary objective is to induce vibration in the flower without making physical contact. This non-contact approach ensures that the delicate floral structures remain undamaged while also minimizing the risk of transmitting diseases between flowers, which is a common concern with manual or mechanical pollination techniques.

### 5.1 Proposed end-effector design

**Figure 8:**
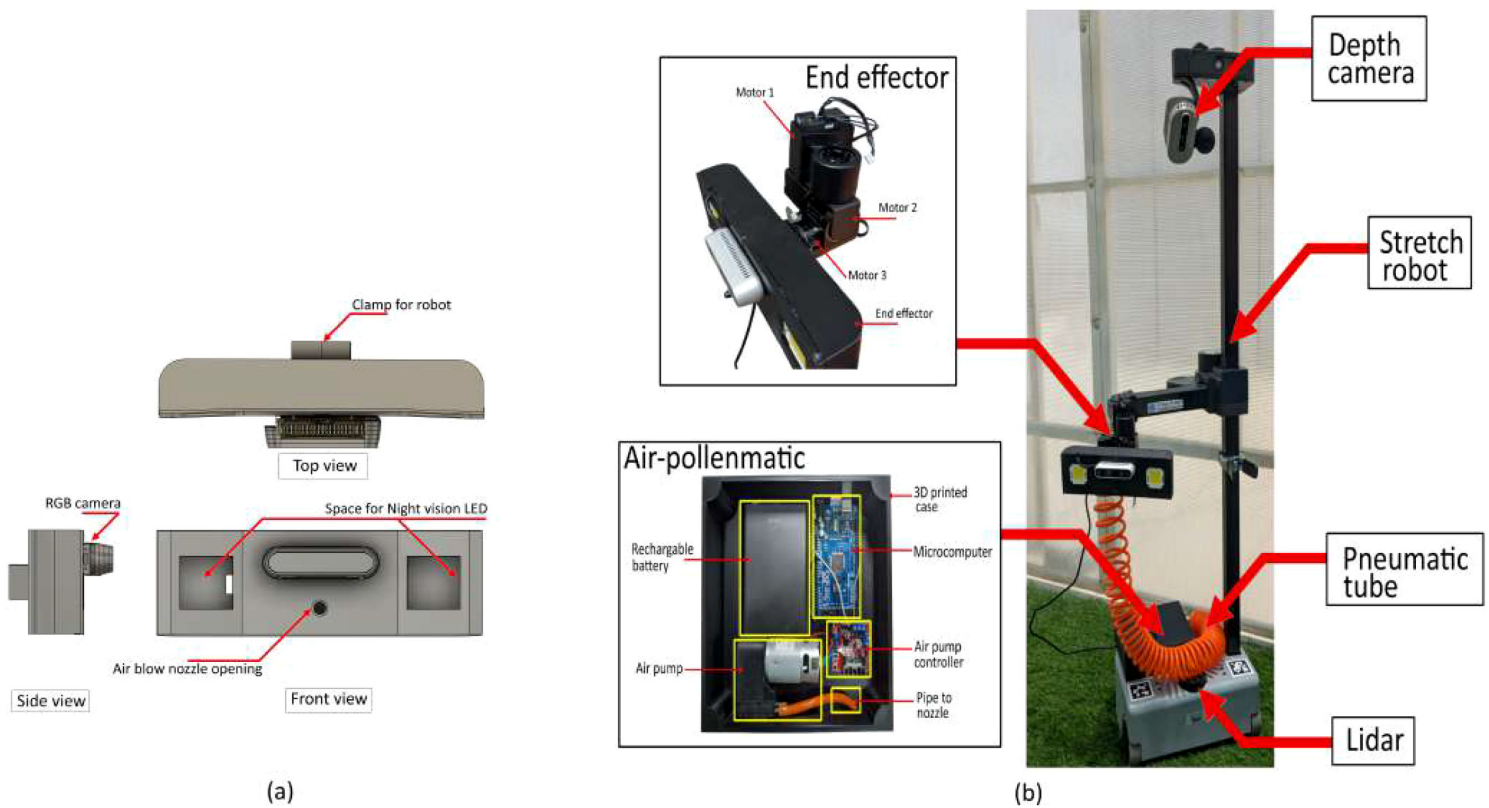
Design of Air Pollenmatic system: **(a)** CAD model design of airflow based pollinator end effector, and **(b)** Stretch robot equipped with Air Pollenmatic system, airflow based end effector, lidar for navigation, RGB-D camera for object detection, and mobile base.

An electronic tomato pollination device called the Air Pollenmatic was initially developed to enable efficient tomato pollination using airflow technology, as illustrated in **Figures 8a, b**. The airflow-based Air Pollenmatic device measures 200 mm × 200 mm × 50 mm and weighs 1.2 kg. Its simple design and compact form factor make it well-suited for non-contact tomato pollination operations. The device operates on a 12 V rechargeable lithium battery that powers an air pump to produce airflow. A cost-effective Arduino Mega microcontroller generates PWM (pulse width modulation) signals to regulate the airflow behavior. **Figures 8a, b** shows the CAD design of the novel non-contact airflow-based pollinator for tomato plants and stretch robot equipped with Air Pollenmatic system. The design incorporates an RGB-D camera (Intel RealSense D435), two 12 V night vision LEDs, and a stainless steel nozzle. The Air Pollenmatic body is fabricated using 3D printed technology. The nozzle is attached to the Air Pollenmatic device using air pipe.

**Figure 8b** demonstrates the integration of the Stretch robot platform for conducting real-time airflow-based pollination operations. The proposed Air Pollenmatic system is seamlessly integrated with the robot’s mobile base, while a specialized airflow-based pollination end-effector is mounted at the robot’s end point to perform pollination functions. The end-effector configuration features three Dynamixel XM450 servo motors arranged in the assembly depicted in **Figure 8b**. This mechanical arrangement emulates human wrist joint kinematics, providing three critical degrees of freedom through yaw, pitch, and roll articulation. The combined rotational capabilities enable the end-effector to achieve precise spatial positioning for comprehensive pollination coverage. The robotic system incorporates a telescopic arm mechanism coupled with a differential drive locomotion base. Environmental perception is achieved through LiDAR sensing and RGB-D camera systems, facilitating obstacle detection and autonomous navigation capabilities.

**Table 5** presents the primary components of the Air Pollenmatic device along with their key specifications.

**Table 5:** Key components and specifications of Air Pollenmatic device.

Main components	Specifications	Values
	Type	Lithium battery
Power Supply	Voltage	12 V
	Cycle life	Up to 5000 cycles
	Air flow rate	40 L min^− 1^
Air pump	Rated Power	40 W
	Voltage	12 V
	Micro-processor	Arduino Mega
Controller	Clock frequency	16 MHz
	Flash memory	256

### 5.2 Deployment on robotic platform

**Figure 9:**
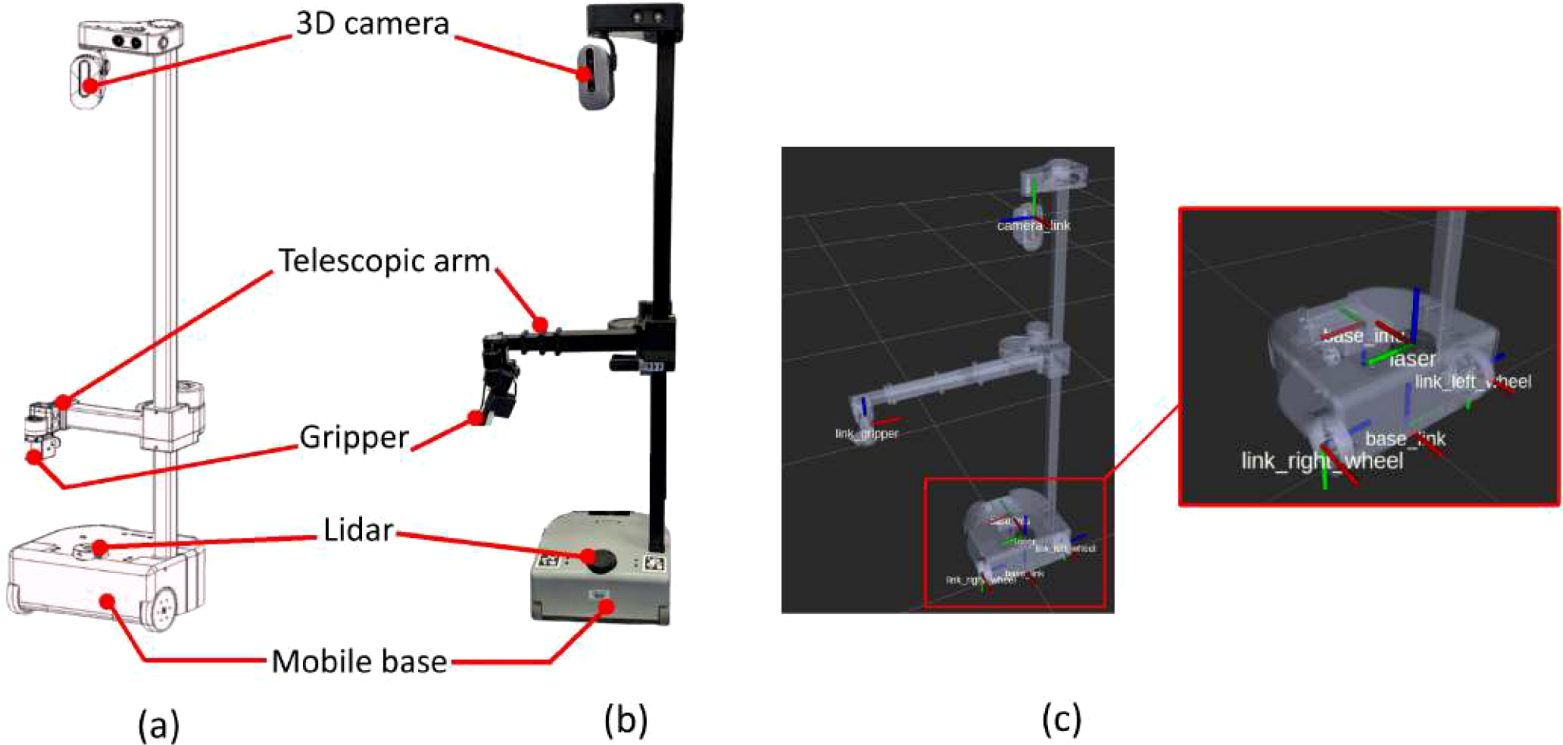
Stretch robot **(a)** CAD model, **(b)** Robot model, and **(c)** ROS model.

The experimental setup utilizes the Stretch robot system (www.hello-robot.com) as the foundational platform for task execution as depicted in **Figure 9**.

Robot Operating System (ROS) Quigley (2009) serves as a robotic middleware, offering software frame-works for the development of robotic software. ROS incorporates numerous open-source implementations of commonly used robotics functionality and algorithms. These implementations are organized into packages within ROS distributions. In our work, we leverage the cutting-edge software tool MoveIt Deng et al. (2017) to facilitate the development of an intuitive workflow for creating robotic applications in the context of tomato flower pollination within a greenhouse environment. By utilizing MoveIt, we ensure an easily accessible and user-friendly approach for developing and implementing robotic applications specific to the task at hand. The integration of MoveIt with ROS allows for seamless control and motion planning, enhancing the efficiency and effectiveness of the robotic system.

The motion planning plugin in MoveIt is built on the Open Motion Planning Library (OMPL) and interfaces with motion planners through ROS Actions or services. OMPL offers core libraries that support the integration of diverse planners with multiple algorithms, ensuring the planner module remains extensible. Through OMPL, planners generate trajectory plans based on specified start and goal configurations while accounting for geometric and kinematic/dynamic constraints. Effective motion planning depends on coordination between the kinematic solver and collision checker components. MoveIt also supports the development of custom inverse kinematic algorithms by leveraging the RobotState class for forward kinematics calculations and Jacobian computation. In this work, we integrated the TracIK inverse kinematics solver within MoveIt, selected for its efficiency and stability. The motion planner produces collision-free paths for the robot operating in a greenhouse environment.

#### 5.2.1 Image guided visual servoing

**Figure 10:**
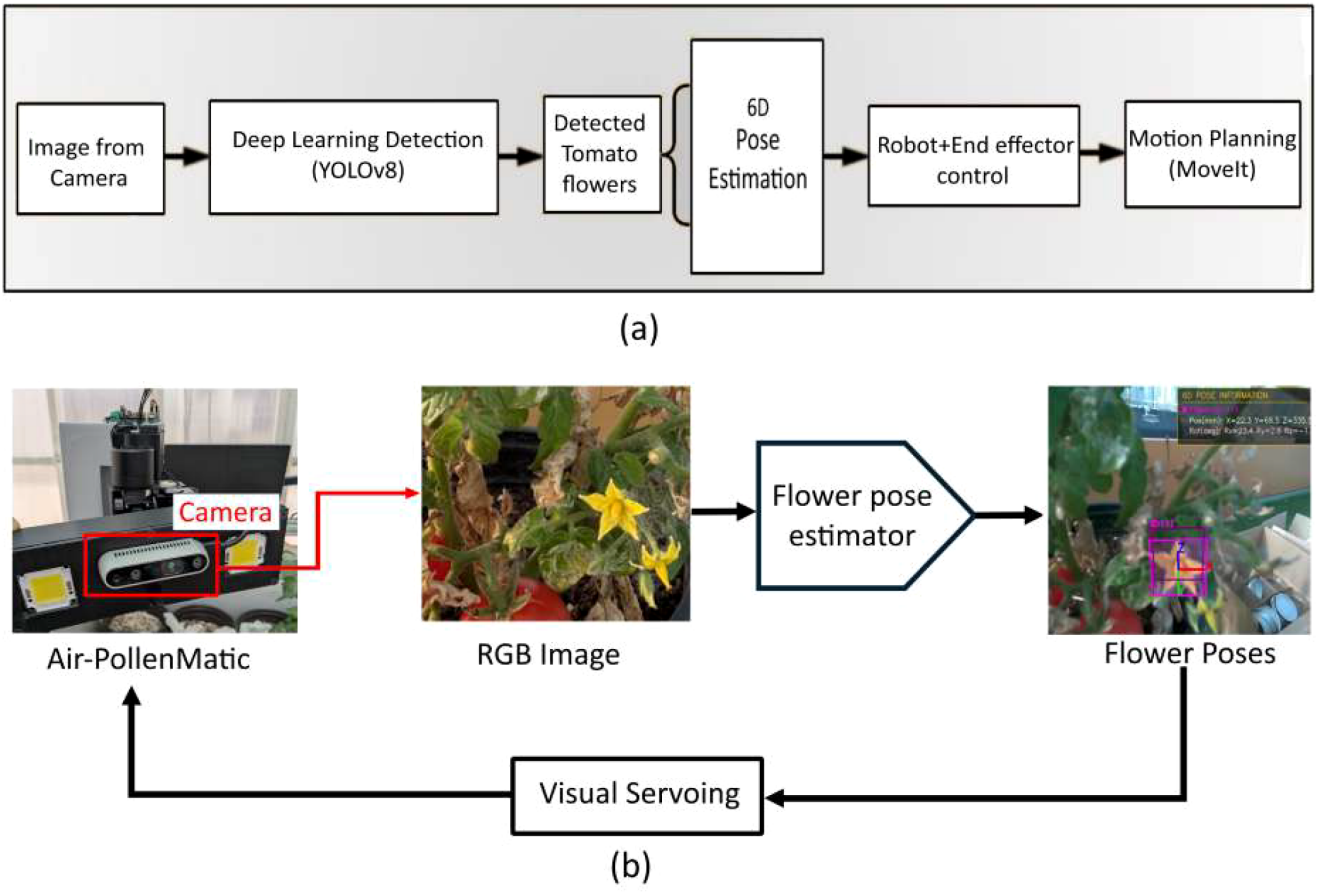
**(a)** System architecture of the robotic pollination pipeline. The workflow begins with image acquisition from the camera, followed by deep learning-based flower detection using YOLOv8. Detected tomato flowers are processed through 6D pose estimation to determine spatial position and orientation. The estimated pose information is then used for robot and end-effector control, with motion planning executed through MoveIt for precise pollinator positioning and **(b)** Autonomous pollination system workflow. The Air Pollenmatic robot uses an onboard camera to capture RGB images of flowering plants. A flower pose estimator processes these images to determine 3D positions and orientations of target flowers, visualized as colored bounding boxes with coordinate frames. The estimated poses feed into a visual servoing system that autonomously guides the robot for precise pollination positioning. This closed-loop approach combines computer vision-based flower detection with robotic control to enable automated agricultural pollination tasks.

**Figure 10a** illustrates the utilization of a deep learning model (YOLOv8) in the camera system to recognize tomato flower. This recognition process enables the identification of flowers and provides valuable visual information, including orientation. These factors play a crucial role in the control loops of the proposed robot control system. In this study, we refer to this methodology as image feedback guided visual servoing, where the visual information guides the robot’s and end effector actions and decision-making processes in coordination with ROS-MoveIt. For tomato flower selection, an RGB-D camera detects the depth of identified flowers using a sensor mounted on the proposed end effector. The selection process prioritizes detections based on their confidence values and associated IDs, with preference given to those exhibiting the highest confidence.

Maintaining proper flower orientation is critical to ensure accurate alignment between the end effector nozzle center and the detected flower, which is essential for effective pollination. To achieve precise end effector positioning for each detected flower, a PnP-based 6D pose estimation model is applied individually to every flower. Flower positions are derived from pixel coordinates obtained through image processing techniques. Concurrently, the RGB-D camera mounted on the end effector captures corresponding depth information for each flower, enabling non-contact pollination with suitable distance (say 3–5 cm).

By integrating the extracted positional data with the estimated depth measurements, the robot achieves accurate flower localization. The visual 6D pose estimation data is converted into ROS node and used to control end-effector motors and robot motors for accurate pose estimation of pollinator (end-effector). This capability allows the robot to navigate efficiently and position its end effector nozzle center precisely at the center of each target flower as depicted in **Figure 10b**.

### 5.3 Experimentation

The integrated hardware platform, featuring the customized Air Pollenmatic system and deployment on the robot, underwent experimental validation within the greenhouse facilities at Khalifa University, Abu Dhabi.

#### 5.3.1 Pollination performance

**Figure 11:**
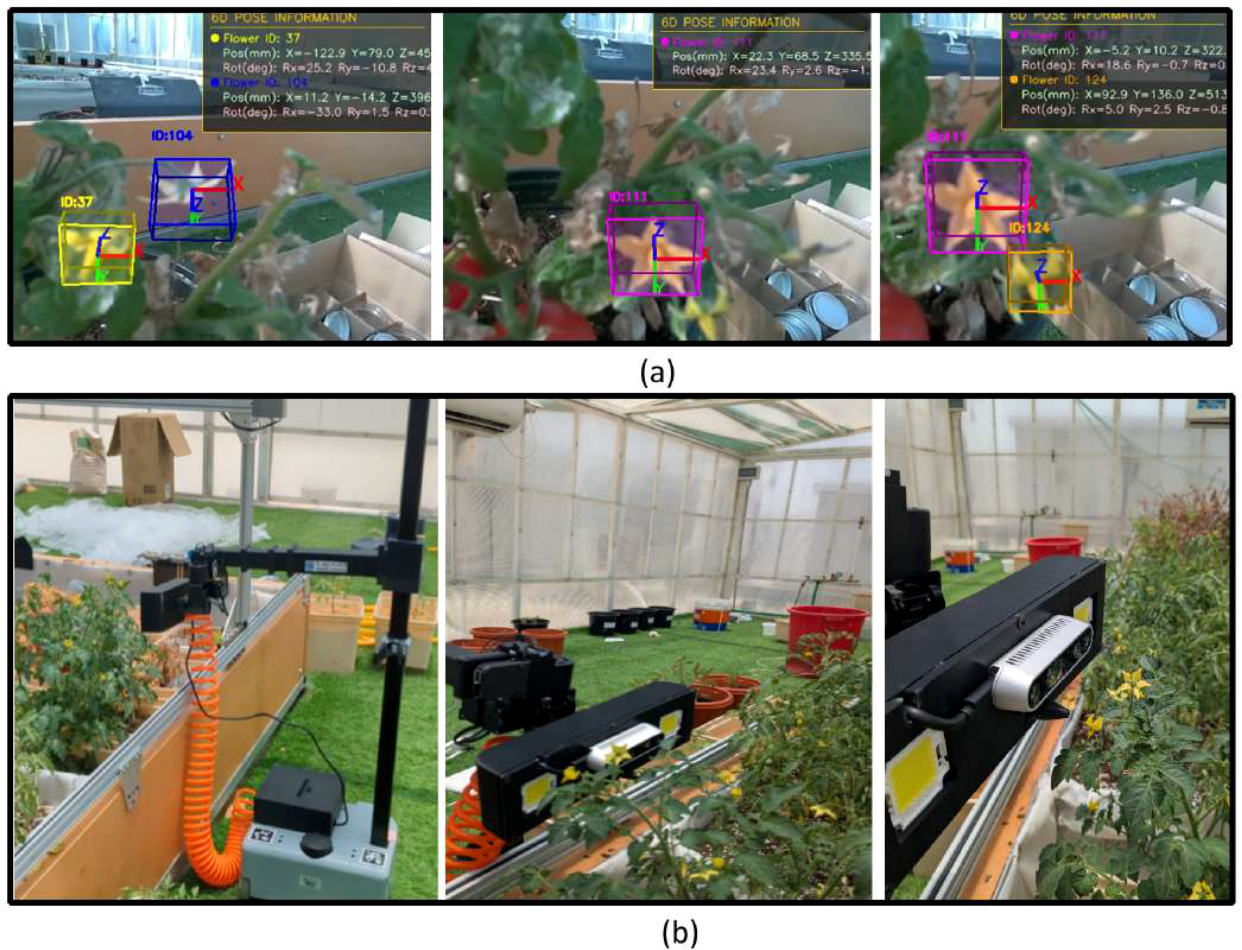
Experimentation validation **(a)** tomato flower detection, assigning IDs, and pose estimation, and **(b)** real greenhouse trails.

Real-world experimental validation was conducted at Khalifa University’s greenhouse facility in Abu Dhabi using a robotic manipulator equipped with eye-in-hand vision and an air-based pollination end-effector. The system demonstrated successful autonomous pollination capabilities by executing a coordinated sequence of single-shot pose estimation, flower detection, Perspective-n-Point pose calculation, and visual servoing control. **Figure 11** showcases the experimental findings from the greenhouse trials: (a) illustrates the detected tomato flowers with assigned IDs and computed poses, while (b) captures the airflow-based pollination system actively performing pollination tasks in the operational greenhouse environment. The experimental methodology utilized two quantitative metrics for performance assessment: pollination attempt rate, representing the count of pollination efforts on reachable flowers, and pollination success rate, denoting the fraction of attempts resulting in successful pollination.

Success criteria encompass proper alignment of the air nozzle with the target flower and subsequent air pulse execution. To establish measurement reliability, each experimental condition was replicated 30 times, with mean performance values summarized in **Table 6**.

**Table 6:** Comparison of contact and non-contact pollination methods.

Metric	Contact Shrestha et al. (2025)	Non-contact	Δ (%)	*p*-value
Attempt Rate	90.15 ± 4.87	92.38 ± 4.12	+2.23	0.042*
Success Rate	78.62 ± 5.94	85.71 ± 4.85	+7.09	*<* 0.001***
Overall Efficacy	70.9	79.2	+8.3	*<* 0.001***

**Table 7** presents the summary statistics from 30 independent trials comparing the performance of the Contact Method and Our Method across two key metrics: Attempt Rate and Success Rate. Our Method demonstrated superior performance in both metrics, achieving a mean Attempt Rate of 92.38% (95% CI: [90.85%, 93.91%]) compared to 90.15% (95% CI: [88.33%, 91.97%]) for the Contact Method, representing a 2.23 percentage point improvement. More notably, Our Method achieved a mean Success Rate of 85.71% (95% CI: [83.90%, 87.52%]), substantially outperforming the Contact Method’s 78.62% (95% CI: [76.40%, 80.84%]), with a 7.09 percentage point improvement. The non-overlapping confidence intervals for Success Rate provide strong evidence of statistically significant improvement. Additionally, Our Method exhibited lower variability across both metrics, with standard deviations of 4.12% and 4.85% for Attempt.

**Table 7:** Summary statistics for pollination performance metrics (30 trials).

Metric	Method	Mean (%)	Std dev (%)	Std error (%)	95% CI
Attempt Rate	Contact Method	90.15	4.87	0.89	[88.33, 91.97]
	Our Method	92.38	4.12	0.75	[90.85, 93.91]
Success Rate	Contact Method	78.62	5.94	1.08	[76.40, 80.84]
	Our Method	85.71	4.85	0.89	[83.90, 87.52]

Rate and Success Rate respectively, compared to 4.87% and 5.94% for the Contact Method, indicating greater reliability and consistency. These results demonstrate that Our Method not only achieves higher performance but also delivers more predictable outcomes, making it a superior approach for pollination performance evaluation.

This indicates that the air-based system can successfully initiate pollination attempts on a higher percent-age of flowers within the manipulator’s operational workspace. More significantly, the success rate of the non-contact method reached 85.6%, substantially outperforming the contact method’s 78.75% success rate by 6.85%. When considering the overall pollination effectiveness, calculated as the product of attempt rate and success rate, the proposed air-based approach achieves 79.2% overall effectiveness compared to 70.9% for the contact method, demonstrating an 8.3% improvement in total system performance. These results validate the practical advantages of the non-contact pollination approach, including reduced physical constraints during flower access, improved operational flexibility, and enhanced reliability in pollination delivery without the risk of flower damage associated with physical contact methods.

**Figure 12:**
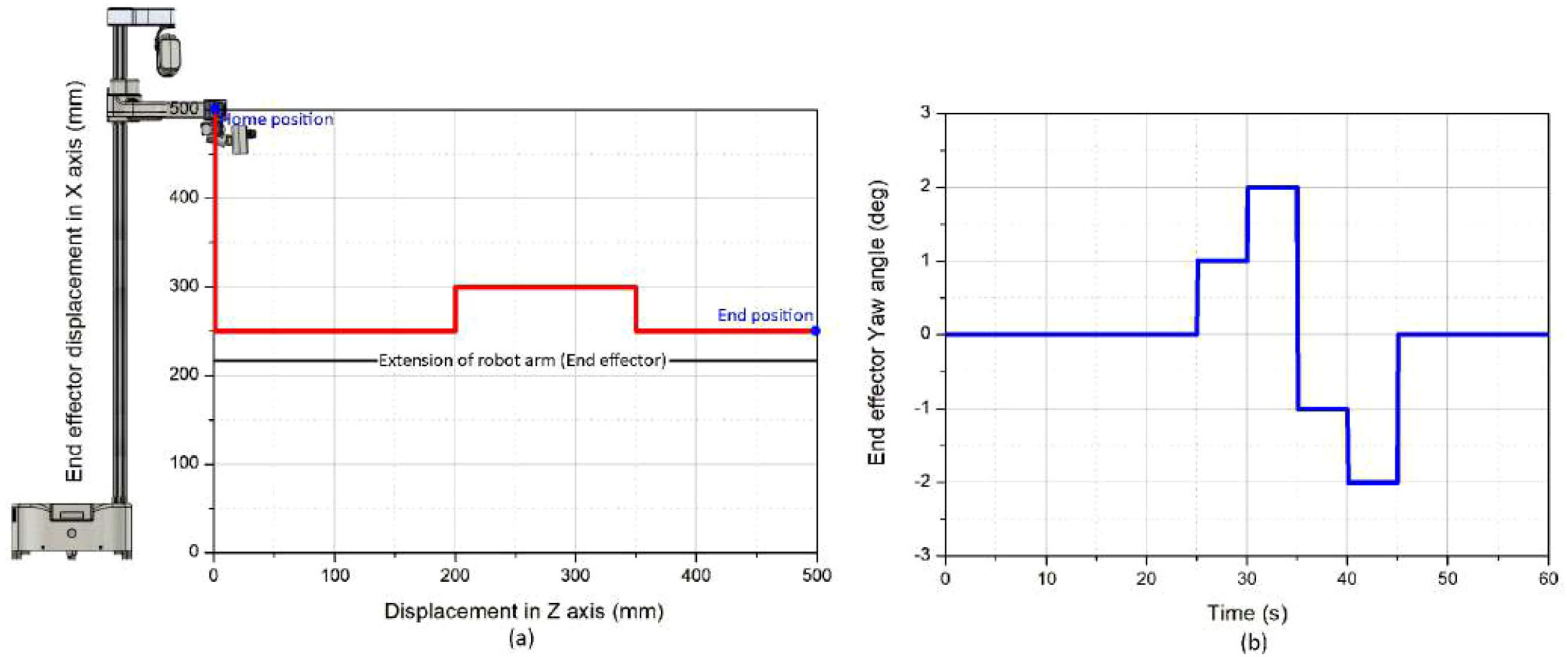
End-effector kinematics during robotic pollination operation. **(a)** Spatial trajectory showing vertical descent and horizontal arm extension in the X–Z plane for multi-flower targeting. **(b)** Yaw angle variation over time demonstrating orientation adjustments via visual servoing control for precise airflow delivery.

**Figure 12** shows the end effector motion trajectory and orientation control during pollination operation. (a) Spatial trajectory of the end effector showing displacement in the X–Z plane. The robot initiates from the home position (blue marker, X = 500 mm, Z = 0 mm), descends vertically to approximately X = 250 mm, then extends horizontally along the Z-axis through a step-wise trajectory reaching the end position (blue marker, Z = 500 mm). The black reference line indicates the robot arm extension path. The step pattern reflects sequential positioning adjustments for targeting multiple flower clusters at different depths. (b) End-effector yaw angle variation over time during the pollination sequence. The yaw angle oscillates between +2 deg and − 2 deg over the 60 second operation cycle, demonstrating the 3 DOF wrist mechanism’s capability to adjust nozzle orientation for optimal airflow alignment with varying flower poses. The angular adjustments correspond to real-time visual servoing corrections based on 6D pose estimation feedback from the YOLOv8-PnP pipeline.

## 6 Discussion

The experimental results presented in Section 5 validate the effectiveness of the proposed non-contact robotic pollination approach. The significant improvements in both detection accuracy and pollination efficacy over baseline methods warrant deeper analysis regarding contributing factors, comparison with existing systems, and implications for practical deployment. The following subsections contextualize these findings within the broader landscape of agricultural robotics research.

### 6.1 Comparison with existing robotic pollination systems

The 79.2% overall pollination efficacy achieved by the proposed non-contact system represents a substantial advancement over existing robotic pollinators reported in the literature. The StickBug six-armed precision pollination robot developed by Smith et al. (2024), which employs contact-based vibrating probes, reported approximately 66% success rates in greenhouse environments—13.2 percentage points lower than our approach. Similarly, the FLOPE (Flower Pose Estimation) system presented by Shrestha et al. achieved 85% pose estimation accuracy for precision pollination applications, whereas our YOLOv8-PnP fusion demonstrates superior detection performance at 95.8% precision. This performance differential can be attributed to the integration of real-time depth information from the Intel RealSense D435 camera and the implementation of RANSAC-based outlier rejection within the PnP solver, which enhances robustness against detection noise and partial occlusions common in dense greenhouse canopies.

Compared to commercial systems such as Arugga’s AI-based pollination robots Arugga (2022), which utilize fixed nozzle configurations for high-wire tomato cultivation, our system offers enhanced adaptability through precise 6D pose estimation that enables dynamic nozzle positioning relative to individual flower orientations. The multi-degree-of-freedom pneumatic system proposed by Masuda et al., while innovative in its soft tube-based extension mechanism, presents installation complexity and bulky design configurations that limit integration with mobile robotic platforms. In contrast, the Air Pollenmatic device (200 mm × 200 mm × 50 mm, 1.2 kg) demonstrates a compact form factor specifically designed for seamless integration with the Hello Robot Stretch platform, enabling autonomous greenhouse navigation during pollination operations.

### 6.2 Detection performance analysis

The detection performance of our YOLOv8-based model surpasses previously reported results for agricultural flower detection tasks. The comparative analysis against baseline architectures revealed consistent superiority: YOLOv8-PnP achieved 97.7% mAP compared to 93.2% for YOLOv5-PnP, 89.6% for ResNet-PnP, and 85.2% for SIFT-PnP approaches. The 4.5 percentage point improvement over YOLOv5 can be attributed to YOLOv8’s enhanced CSPDarknet53 backbone architecture and improved feature pyramid network (FPN) with path aggregation network (PAN) for multi-scale feature fusion Jocher et al. (2023). Furthermore, the real-time processing capability at 28.5 FPS exceeds the 22 FPS achieved by YOLOv5-PnP and the 16 FPS of ResNet-PnP, making the proposed system suitable for continuous autonomous operation in commercial greenhouse settings where processing speed directly impacts operational throughput. Notably, existing fruit detection and pose estimation methods developed for harvesting applications present fundamental limitations when applied to flower pollination tasks. Fruit pose estimation approaches such as those developed for apple harvesting Ji et al. (2012) and citrus picking Hwang et al. (2024) typically rely on distinct geometric features including spherical or ellipsoidal shapes, uniform color distributions, and rigid surface characteristics. In contrast, tomato flowers present significantly more challenging detection scenarios characterized by: (1) non-rigid, deformable petal structures that vary with environmental conditions and developmental stage; (2) small target sizes (typically 15–25 mm diameter) compared to mature fruits;(3) dense clustering patterns where multiple flowers share overlapping visual boundaries; and (4) color similarity with surrounding foliage under varying illumination Dingley et al. (2022); Tang et al. (2020). Furthermore, keypoint-based pose estimation methods successfully applied to tomato peduncle detection Ci et al. (2024) and strawberry harvesting Li and Kasaei (2024) assume consistent anatomical landmarks visible from the camera perspective. However, the radially symmetric structure of tomato flowers with variable petal orientations creates ambiguous keypoint correspondences, particularly when flowers are partially occluded by adjacent blooms or foliage. The proposed YOLOv8-PnP fusion addresses these challenges through bounding box-based pose estimation that does not require explicit keypoint visibility, combined with RANSAC outlier rejection to handle detection noise inherent in cluttered greenhouse environments.

### 6.3 Advantages of non-contact pollination

The superior performance of the non-contact approach stems from three key factors. First, eliminating mechanical interference prevents flower displacement and tissue damage during pollination attempts—a critical consideration given that contact-based end-effectors designed for fruit manipulation (such as suction cups and soft grippers) can cause irreversible damage to delicate floral structures Arad et al. (2020); Xiong et al. (2020). Second, the airflow cone’s spatial coverage relaxes positioning tolerance requirements compared to point-contact probes requiring sub-centimeter accuracy, accommodating inherent visual servoing errors while maintaining effective pollen release stimulation Liu et al. (2025). This tolerance relaxation is particularly important because flower pose estimation inherently exhibits higher uncertainty than rigid fruit localization due to the factors discussed above. Third, the non-contact methodology eliminates pathogen transmission pathways between plants, addressing a critical biosecurity concern in protected cultivation environments where diseases such as Botrytis cinerea can spread rapidly through physical contact vectors Williamson et al. (2007); Elad and Pertot (2014). Additionally, unlike fruit harvesting where end-effector contact with the target is the operational objective, pollination requires inducing mechanical vibration without displacement—a fundamentally different manipulation paradigm that existing fruit-handling approaches cannot accommodate Singh et al. (2025a). The airflow-based actuation employed in this study provides precisely this capability, generating the 240–400 Hz vibrational frequencies required for pollen release Dingley et al. (2022) without the positioning precision demands of physical grasping operations.

### 6.4 System design considerations

The modular design philosophy—separating the Air Pollenmatic device from the mobile platform 643 through ROS-based interfaces—enables deployment flexibility across diverse robotic systems and facilitates technology transfer to different greenhouse configurations. The compact end-effector design and 3-DOF wrist mechanism provide sufficient dexterity for omnidirectional pollination coverage while maintaining the payload constraints of lightweight mobile manipulators.

### 6.5 Limitations and future directions

Several research directions emerge to advance autonomous pollination toward commercial viability. First, developing adaptive airflow control mechanisms that dynamically adjust pressure, flow rate, and pulse duration based on real-time flower phenotype assessment would improve pollination consistency. High-speed imaging for visualizing pollen release could enable closed-loop feedback optimization. Second, expanding perception to incorporate multimodal sensing—combining RGB-D data with thermal imaging for flower receptivity assessment and pollen release detection—would enhance decision making for optimal pollination timing. Finally, extending applicability to other crops such as peppers, eggplants, and strawberries would broaden commercial impact, leveraging the transferable YOLOv8-PnP framework.

## 7 Conclusions

This study establishes the technical feasibility and performance advantages of non-contact airflow-based robotic pollination for greenhouse tomatoes. The integration of state-of-the-art deep learning detection with precision pose estimation and visual servoing control achieves pollination efficacy exceeding current robotic alternatives while offering inherent advantages in flower preservation and disease prevention. The proposed YOLOv8-PnP hybrid architecture demonstrated exceptional detection performance with 95.8% precision and 97.7% mAP@0.5, enabling real-time flower localization at 28.5 FPS suitable for autonomous greenhouse operations. Field validation confirmed that the Air Pollenmatic system achieved 79.2% overall pollination efficacy, representing an 8.3 percentage point improvement over contact-based methods with statistically significant performance gains (*p <* 0.001). While limitations in dataset diversity, environmental generalization, and long-term agronomic outcome validation remain to be addressed through extended field trials, the demonstrated performance improvements provide a solid foundation for continued development toward commercially deployable autonomous pollination systems. The modular, ROS-based system architecture ensures adaptability across different robotic platforms and greenhouse configurations, facilitating broader adoption of precision pollination technology in protected agriculture. 

## Data Availability

The raw data supporting the conclusions of this article will be made available by the authors, without undue reservation.
